# Uncovering unsuspected advanced liver fibrosis in patients referred to alcohol nurse specialists using the ELF test

**DOI:** 10.1186/s12876-021-01728-2

**Published:** 2021-03-31

**Authors:** Freya Rhodes, Sara Cococcia, Jasmina Panovska-Griffiths, Sudeep Tanwar, Rachel H. Westbrook, Alison Rodger, William M. Rosenberg

**Affiliations:** 1 Institute for Liver and Digestive Health, Division of Medicine, University College London, Royal Free Campus, Rowland Hill Street, Hampstead, London, NW3 2PF UK; 2 First Department of Internal Medicine, San Matteo Hospital Foundation, University of Pavia, Pavia, Italy; 3 Department of Applied Health Research, University College London, London, UK; 4 Institute for Global Health, University College London, London, UK; 5 Barts Health NHS Trust, London, UK; 6 Institute for Liver and Digestive Health, UCL Division of Medicine, Royal Free Campus, London, NW3 2QG UK

**Keywords:** Liver cirrhosis, Liver diseases, Alcoholic, Non-invasive test, Alcohol use disorder, Enhanced liver fibrosis test

## Abstract

**Background and aims:**

Alcohol use disorders (AUD) cause 7.2% of UK hospital admissions/year. Most are not managed by hepatologists and liver disease may be missed. We used the Enhanced Liver Fibrosis (ELF) test to investigate prevalence and associations of occult advanced liver fibrosis in AUD patients not known to have liver fibrosis.

**Methods:**

Liver fibrosis was assessed using ELF in prospective patients referred to the Royal Free Hospital Alcohol Specialist Nurse (November 2018–December 2019). Known cases of liver disease were excluded. Patient demographics, blood tests, imaging data and alcohol histories recorded. Advanced fibrosis was categorised as ELF ≥ 10.5.

**Results:**

The study included 99 patients (69% male, mean age 53.1 ± 14.4) with median alcohol intake 140 units/week (IQR 80.9–280), and a mean duration of harmful drinking of 15 years (IQR 10–27.5). The commonest reason for admission was symptomatic alcohol withdrawal (36%). The median ELF score was 9.62, range 6.87–13.78. An ELF score ≥ 10.5 was recorded in 28/99 (29%) patients, of whom 28.6% had normal liver tests. Within previous 5-years, 76% had attended A&E without assessment of liver disease. The ELF score was not associated with recent alcohol intake (*p* = 0.081), or inflammation (*p* = 0.574).

**Conclusion:**

Over a quarter of patients with AUD had previously undetected advanced liver fibrosis assessed by ELF testing. ELF was not associated with liver inflammation or recent alcohol intake. The majority had recent missed opportunities for investigating liver disease. We recommend clinicians use non-invasive tests to assess liver fibrosis in patients admitted to hospital with AUD.

**Supplementary Information:**

The online version contains supplementary material available at 10.1186/s12876-021-01728-2.

## Highlights

- ¼ patients in hospital with AUD had ELF scores ≥ 10.5 indicative of advanced fibrosis.
- ¾ patients with AUD had attended A&E within 5yrs without liver fibrosis assessment
- ELF score was not associated with inflammation (AST or ALT)
- ELF score was not associated with amount of alcohol consumed (Units/Week)
- 28% of those with advanced fibrosis (ELF ≥ 10.5) had normal liver function tests

## Background

One in five people in the UK drink alcohol at hazardous or harmful levels [1]. While alcohol causes a wide array of health and social harms, the greatest morbidity and mortality are associated with alcohol-related liver disease (ArLD) with mortality rates increasing 400% since 1970 [2].

Hospital admissions related to alcohol are rising annually, with 350,000 alcohol related admissions per year in 2019, (an increase of 20% in a decade) [3] and with a cost to the NHS of £3.5 billion per year [4]. This is likely to be due to a shift in drinking behaviours from low-strength beer in pubs to home consumption of higher strength beer, wine and spirits sold in supermarkets. In addition, alcohol is now 64% more affordable than it used to be 30 years ago [1].

While a proportion of people admitted to hospital with harms arising from their drinking behaviour are recognised to have liver fibrosis and are managed by liver specialists, many are managed by a wide range of doctors and their liver disease may be missed, even if their alcohol use disorder (AUD) is recognised.

Moreover, it is estimated that up to 75% of people with Chronic Liver Disease (CLD) first present to healthcare when their liver disease is advanced often with decompensated cirrhosis, when it is too late for behaviour change or interventions to avert poor outcomes [1, 5, 6].

Part of the reason for this is because cirrhosis is often asymptomatic, and it is not reliably detected using routine liver function blood tests (LFTs) or ultrasound [7]. The last two decades have witnessed the development and validation of a number of non-invasive tests for liver fibrosis that are increasingly used in clinical practice. These tests create the possibility to detect advanced liver fibrosis in at-risk patients with AUD, in order to refer them appropriately to hepatology services for regular follow up to avert, or detect and treat complications of portal hypertension including oesophageal varices [8, 9] and ascites [10], and screening for liver cancers [11, 12], as well as alcohol counselling from hospital or community services as appropriate.

Currently non-invasive testing for liver fibrosis with the Enhanced Liver Fibrosis test (ELF) is widely used for Non-Alcoholic-Fatty Liver Disease (NAFLD) to determine which patients with fatty liver have advanced fibrosis and warrant referral to hepatology, versus those with low ELF scores who can remain in primary care [13]. However, although evidence-based [14] and recommended by BSG guidance, [7] this approach is not yet in widespread use in the NHS for people with AUD.

We aimed to investigate the prevalence of advanced liver fibrosis using the ELF test employing the literature-based ELF cut-off of 10.5 [14], in patients recognised to have AUD but not recognised as having liver disease. In addition, we aimed to examine the relationship between demographic factors (including age, Body Mass Index (BMI), deprivation score, baseline LFTs and the ELF score.

## Methods

### Study design

This was a prospective service evaluation conducted at the Royal Free Hospital from November 2018 to December 2019.

### Patient population

Consecutive referrals to the Royal Free Hampstead alcohol specialist nurse (ASN) were included if aged ≥ 18 and excluded if already under the care of a hepatologist, if they had a known chronic liver condition, a diagnosis of alcoholic hepatitis, or acute liver injury secondary to a cause other than alcohol. Out of 100 consecutive referrals to the ASN 98 were in-patients or emergency department attendees. The vast majority of out-patient referrals were ineligible for inclusion because they had known ArLD, leaving 2/100 eligible out-patient referrals. One patient was excluded from the analysis as they were found to have an ALT of 1,023 following a pregabalin overdose, reducing the sample size from 100 to 99.

### Clinical data

Data extracted from patients’ electronic medical records included patient demographics, reason for presentation to hospital, BMI, alcohol intake (detailed in next section), postcode to enable deprivation score calculation, results of any imaging or fibrosis tests performed within 6 months of referral to ASN, blood test results to enable calculation of FIB4, AST:ALT ratio and APRI [14], and number of hospital presentations within the last five years.

Data were anonymised and entered into a password protected spreadsheet held on a secure NHS computer.

### Alcohol data

Current alcohol consumption was recorded in units per week (U/w), and duration of ‘excess alcohol consumption’ in years. This was obtained from patients’ self-reported consumption extracted from free text in clinical notes. AUDIT scores were not available. It was noted if the patient had been actively drinking up to the point of presentation to hospital.

### ELF score

An ELF test was performed on consecutive eligible patients referred to the ASN. Serum was extracted from 5 mL blood per patient which was analysed at the Central ELF laboratory (iQur Limited, London). The samples were analysed for HA, PIIINP and TIMP1 levels using the proprietary assays developed by Siemens Healthineers Inc (Tarrytown, New York, USA) for the ELF test, on a Siemens ADVIA centaur^®^ immunoassay system. ELF Scores were calculated from test results using the manufacturer’s published algorithm. An ELF threshold of 10.5 was pre-selected for detection of advanced fibrosis based on recommendations by Thiele et al. [14].

### Outcomes

The primary outcome was the proportion of patients referred to the ASN at the Royal Free Hospital who had previously undetected advanced fibrosis as determined by an ELF score of ≥ 10.5 [14]. Secondary outcomes investigated potential risk factors for advanced fibrosis including alcohol consumption, BMI, age, sex, deprivation score and smoking status. In addition, missed opportunities for diagnosis of liver disease were assessed by counting the number of attendances to hospital within the previous five years without assessment for liver fibrosis.

### Follow up

Patients with ELF scores ≥ 10.5 were sent a letter inviting them to attend a hepatology outpatient clinic to see a hepatologist with an interest in ArLD, and to have a FibroScan. Blood samples were also taken to screen for viral, immunological and metabolic causes of liver disease in accordance with current protocols if these tests had been omitted during their hospital admission.

### Sample size

As this is an exploratory investigation, following statistical advice we accepted a precision of estimate at 0.1 which would generate a sample size of between 62 and 89 using literature-based estimates of prevalence of advanced fibrosis. A post-hoc sample size calculation for 0.29 prevalence of advanced fibrosis, using a precision of estimate at 0.1 and 0.95% CI results in a minimum required sample size of n = 80.

### Statistical analysis

Demographic information was described using frequencies and percentages for categorical variables. Continuous data were described using means and SD or medians and IQR, depending on the normality of the data.

For the comparison of categorical variables, Chi-Squared or, if sample size was less than 5, Fisher’s exact test was used as a conventional test, and for the continuous data the Mann Whitney U (for non-parametric data) or Student’s *t *test for normally distributed data.

Alcohol ‘units per week’ was analysed both as continuous data, and in quartiles. After univariate analyses, to determine the variables associated with the presence of advanced fibrosis with the most significance, a multiple binary logistic regression analysis model was used, using the literature-based ELF threshold of 10.5 [14] and multiple linear regression was used for continuous ELF scores. Variables were selected if they were established in the literature as risk factors for liver fibrosis, and if they had *p* values less than 0.25 in univariate analyses (either against ELF < / ≥ 10.5 or as a continuous variable). All *p* values were 2-sided and they were considered significant if *p* < 0.05. All data were analysed using SPSS software (Version 25.0. Armonk, NY: IBM Corp).

## Results

### Study demographics

The analysis included 99 patients (69% male) with a mean age of 53.1 years (SD 14.4) (Table 1). Average BMI was 26.52 kg/m^2^ (SD 5.94) and 84% were current or past smokers. Alcohol intake was high with a median consumption of 140 U/w (80.9–280), and in this cohort men and women drank similar amounts (*p* = 0.73). The two patients seen in the ASN outpatient clinic had not been drinking alcohol within the past 3 months, and one inpatient had stopped drinking three weeks prior to hospital admission. All of the other 96/99 patients were drinking alcohol up to the point of presentation to hospital. The median duration of alcohol consumption was 15 years (IQR 10–27.5). This cohort of patients were from deprived areas, with 69% of them positioned within the lowest 4 deprivation deciles.

**Table 1:** Comparison of clinical characteristics of patients with and without advanced fibrosis (as determined by ELF score of ≥ 10.5)

Patient characteristics	Overall (n = 99)	Advanced fibrosis	Non-advanced fibrosis	*p* value
		ELF ≥ 10.5	ELF < 10.5	
Age mean sd	53.11 ± 14.37	55.7 ± 12.6	52.1 ± 15	0.266
Male sex n (%)	68/99 (69)	19/28 (68)	49/71 (69)	0.911
BMI mean sd	26.52 ± 5.94	26.4 ± 5.7	26.6 ± 6.1	0.903
T2DM diagnosis (%)	10/99 (10.1%)	4/28 (14.3%)	6/71 (8.5%)	0.46
Smoking status n (%)				
Non-smoker	15 (16)	6 (21)	9 (13)	0.35
Smoker	69 (73.4)	16 (57)	53 (75)	0.1
Ex-smoker	10 (10.6)	5 (18)	5 (7)	0.14
Unknown	5 (5)	1 (4)	4 (6)	
Ongoing active drinking n (%)^a^	96/99 (97)	26/28 (93)	70/71 (99)	0.19
Current alcohol intake U/w, median (IQR)	140 (80.9–280)	112 (70–210)	150 (105–280)	0.031
Years of harmful drinking median (IQR)	15 (10–27.5)	20 (10–28)	15 (7.5–28)	0.357
Signs of CLD on exam				
Yes n (%)	4 (4%)	4 (14.3)	0 (0%)	0.005
No n (%)	95 (96%)	24 (85.7)	71 (100%)	
Abnormal LFTs at referral^b^				
Yes	63 (66.3%)	18 (64.3)	45 (63.4%)	0.712
No	32 (33.7%)	8 (28.6)	24 (33.8%)	
N =	95	26	69	
ALT IU/L median (IQR)	39 (21–73)	38 (14–76)	41 (22–71.75)	0.792
AST IU/L median (IQR)	43 (24–86.5)	52 (21–149)	40 (25.5–79.5)	0.493
MCV IU/L median (IQR)	96.9 (91.2–100.5)	97.8 (91.8–101.7)	96.8 (91.2–99.8)	0.522
Platelet count × 10^9^/L median (IQR)	206.5 (129–271)	203 (101–303)	206.5 (133.3–262.5)	0.645
Bilirubin μmol/L median (IQR)	10 (4–16)	10 (4–20)	10 (4–15.5)	0.624
FIB4 median (IQR)	2.00 (0.94–3.61)	2.04 (1.05–7.6)	1.96 (0.88–3.38)	0.302
APRI median (IQR)	0.64 (0.3–2.08)	0.64 (0.28–2.71)	0.63 (0.3–1.9)	0.552
AST:ALT ratio median (IQR)	1.3 (0.87–1.71)	1.5 (1.0–2.16)	1.26 (0.8–1.6)	0.074
HA median (IQR)	72.1 (35.1–144.5)			
PIIINP median (IQR)	8.18 (5.77–12.94)			
TIMP1 median (IQR)	265.7 (198.6–364)			
ELF median (IQR)	9.62 (8.93–10.6)			
ELF range (lowest to highest)	(6.87–13.78)			

BMI = Body Mass Index, U/w = units per week, CLD = Chronic Liver Disease, T2DM = Type 2 Diabetes Mellitus, ALT = Alanine aminotranferase, AST = Aspartate aminotransferase, MCV = Mean Corpuscular Volume, APRI = AST to platelet ratio index, HA = Hyaluronic acid, PIIINP = Procollagen 3 N-terminal Peptide, ELF = Enhanced Liver Fibrosis score

^a^At time of presentation to hospital or alcohol clinic

^b^Abnormal LFTs defined as raised transaminases or ALP + GGT (Not including isolated hyperbilirubinaemia (Gilbert’s))

### Reasons for presentation to healthcare

**Fig. 1:**
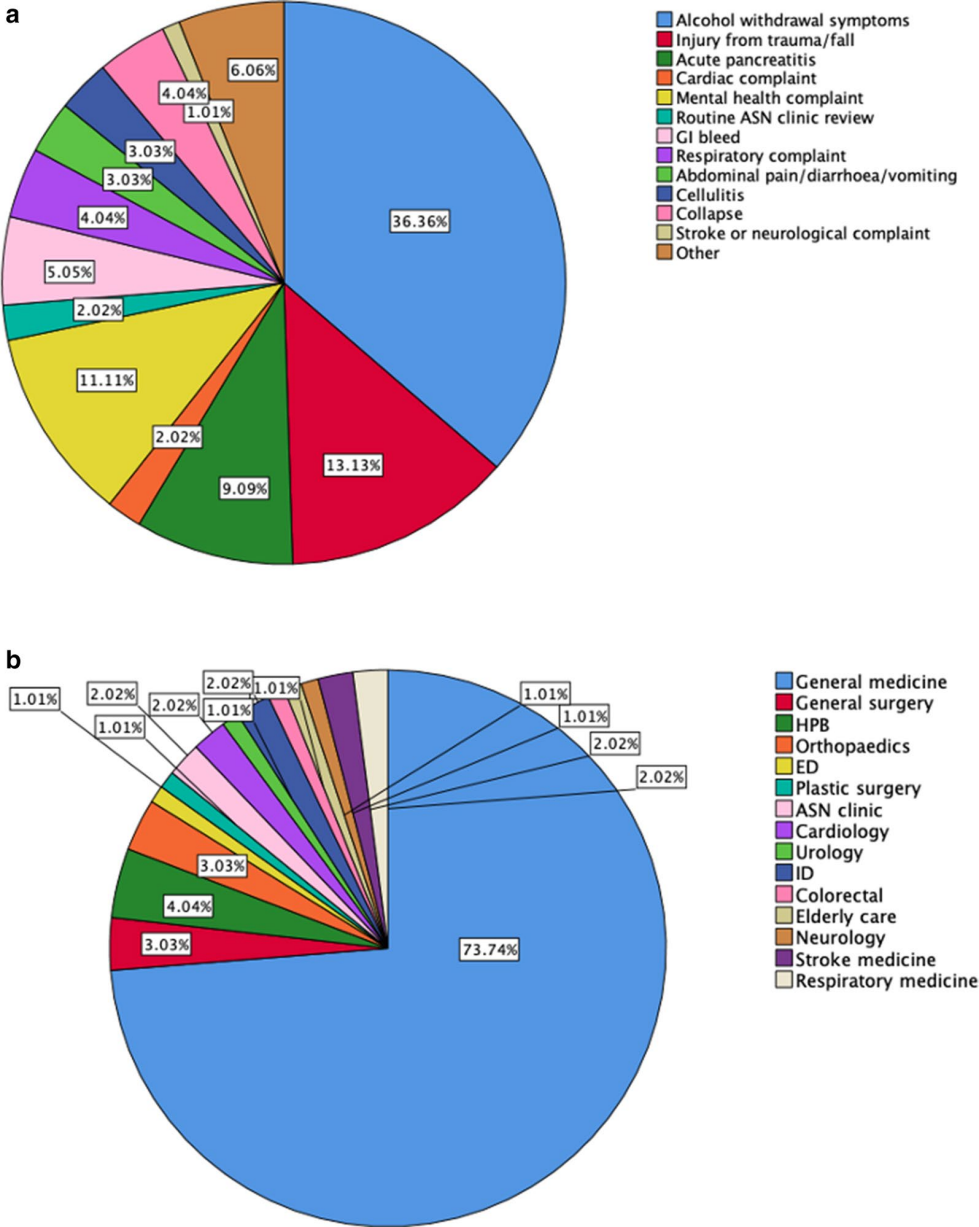
**a** Pie chart of reasons for presentation to hospital. **b** Pie chart of hospital specialty team (AUD = Alcohol Use Disorder, ASN = Alcohol Specialist Nurse, GI = Gastro-Intestinal, HPB = Hepato-Pancreato-Biliary, ED = Emergency Department, ID = Infectious Diseases)

The vast majority (n = 97/99, 98%) of patients were seen as inpatients or in the emergency department. The most common reason for presentation to hospital was symptomatic alcohol-withdrawal (36.4%) including seizures, followed by injuries from falling over (13.1%) and mental health presentations (11.1%) including overdose. The vast majority (73.7%) were under the care of a general medical team (Fig. 1a, b). In the preceding 5 years 76% of the patients had attended hospital without being diagnosed as having ArLD (aside from the current visit), with median number of hospital attendances being 4 (IQR 2–9).

### Results of non-invasive fibrosis tests and LFTs

LFTs were performed in 95/99 patients of which 66.3% of these patients had abnormal LFTs (raised transaminases or ALP + GGT), with median ALT of 39 (21–73) and AST of 43 (24–86.5) (see Table 1). The median ELF score in the whole cohort was 9.62 (IQR 8.93–10.6, range 6.87–13.78). The ELF scores did not differ significantly between men and women (*p* = 0.435).

**Fig. 2:**
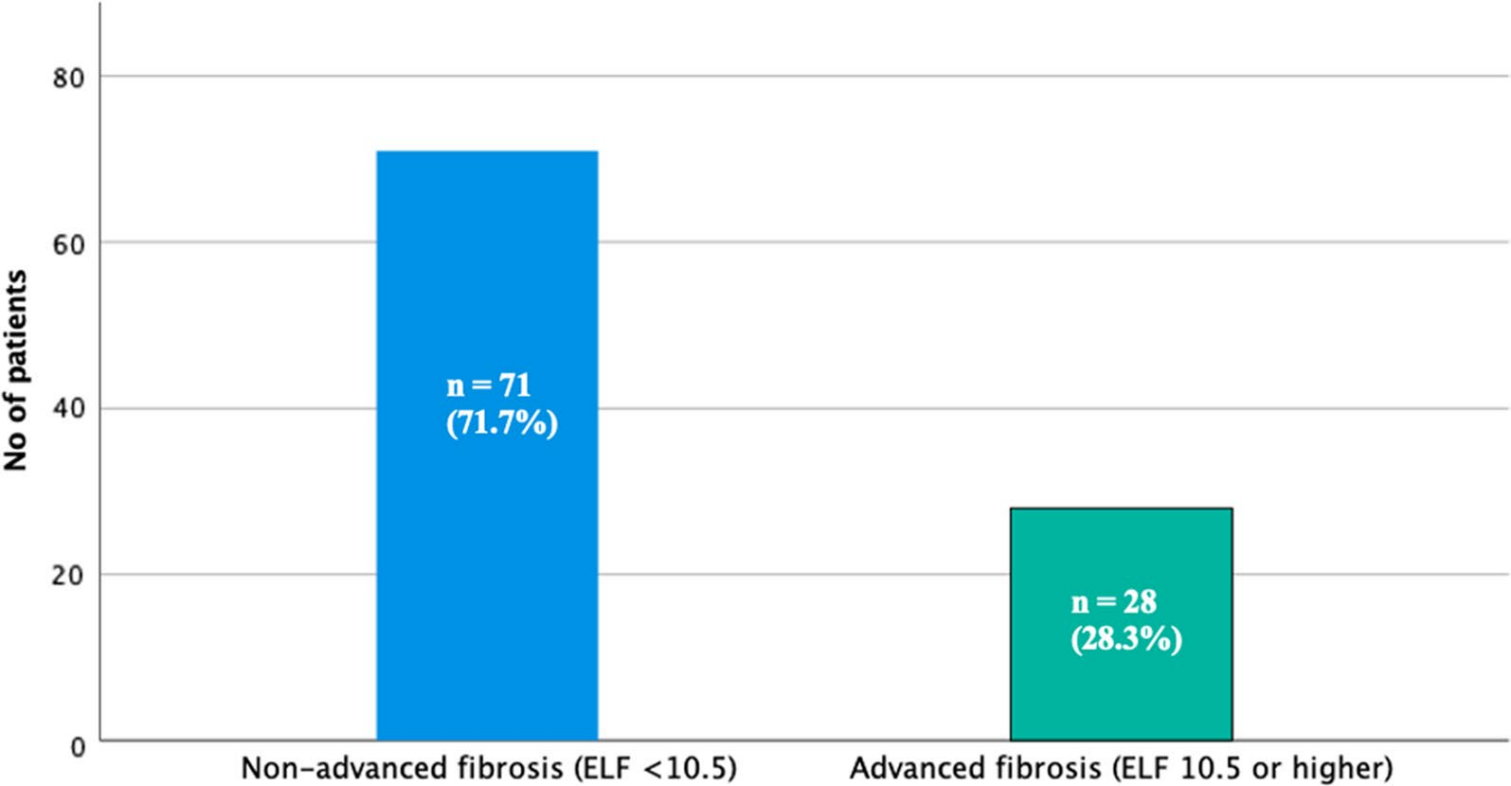
Proportion of patients in study cohort with advanced fibrosis as assessed by ELF ≥ 10.5

Twenty-eight participants (28.3%) had an ELF score of ≥ 10.5, indicating advanced fibrosis (Fig. 2). Of the 28 patients with advanced fibrosis (ELF ≥ 10.5), 8 (28.6%) had normal LFTs.

### Risk factors for high ELF score

Clinical characteristics and baseline LFTs were first compared between patients without and with advanced fibrosis on the basis of ELF score (< 10.5 v ≥ 10.5). In addition, correlations were investigated between these same characteristics and ELF as a continuous variable.

### Comparison of clinical characteristics between patients with and without advanced fibrosis (ELF ≥ 10.5)

**Fig. 3:**
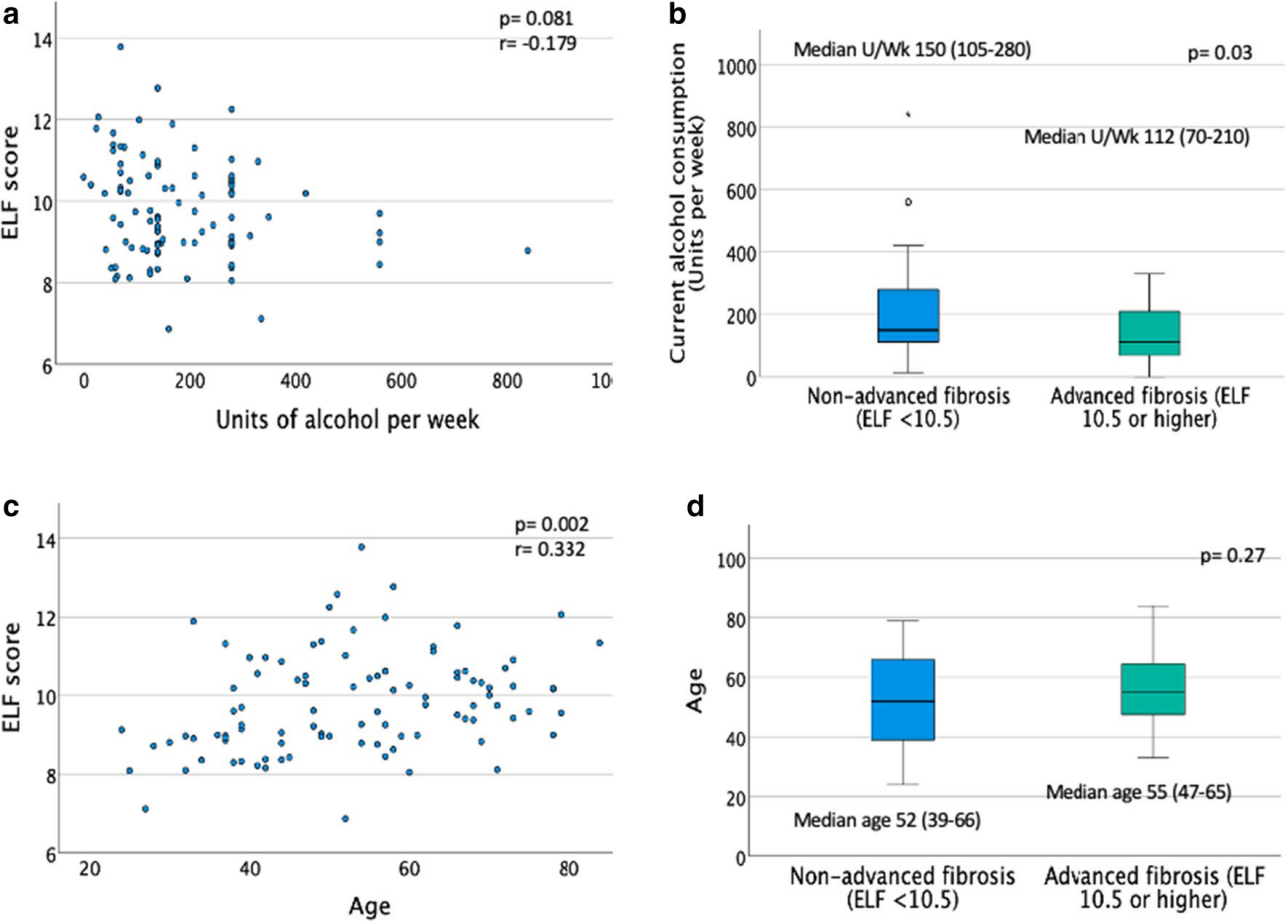
Influence of alcohol and age on binary and continuous ELF scores. A: Scatter plot of ELF by alcohol units per week (Spearman Rho correlation, with *p* value significance set at 0.05, r = correlation coefficient). B: Boxplot of alcohol consumption (Units per week) by presence or absence of advanced fibrosis (ELF ≥ 10.5). Statistical test: Mann Whitney U, *p* value significance set at 0.05, median units per week displayed with IQR (interquartile range). C: Scatter plot of ELF by age (Spearman Rho correlation, with *p* value significance set at 0.05, r = correlation coefficient). D: Boxplot of age by presence or absence of advanced fibrosis (ELF ≥ 10.5). Statistical test: Mann Whitney U, *p* value significance set at 0.05, median age displayed with IQR (interquartile range)

When comparing clinical characteristics with ELF score < 10.5 versus ≥ 10.5 there was no significant difference in age, sex, or BMI between the two groups (Table 1, Fig. 3d). There was also no significant difference in transaminase results, FIB4, or APRI scores between groups. Clinical signs of CLD were only found in patients with ELF score ≥ 10.5 (n = 4). Patients in the advanced fibrosis group (≥ 10.5) drank less alcohol than those with lower ELF scores (mean 112 U/w, compared with 150 U/w, *p* = 0.031, Fig. 3b). However, there was no correlation observed between alcohol consumption and ELF score viewed as a continuous variable (Fig. 3a). Furthermore, multivariable regression analysis revealed no association between alcohol consumption and ELF score (Table 2). There was no difference in the reported duration of alcohol excess in patients with ELF < 10.5 compared to patients with ELF > 10.5 (15 years (10–27.5 years) compared to 20 years (10–28 years); *p* = 0.357).

**Table 2:** Factors associated with advanced fibrosis (ELF ≥ 10.5), as determined by univariable and multivariable regression analyses

Variable	B (unstandardized regression coefficient)	Univariable OR (95% CI)	*p* value	Multivariable OR (95% CI)	*p* value
Age^a^	0.018	1.018 (0.987–1.050)	0.264	1.010 (0.972–1.049)	0.609
Sex (male)	0.054	1.055 (0.412–2.699)	0.911		
BMI	− 0.007	0.993 (0.891–1.107)	0.900		
Current alcohol intake (U/Wk)	− 0.005	0.995 (0.991–1.000)	0.041	0.995 (0.990–1.000)	0.070
Duration of alcohol excess	0.009	1.009 (0.975–1.044)	0.613		
Deprivation score	0.000	1 (1–1)	0.323		
Smoking (non-smoker)	− 0.631	0.532 (0.170–1.667)	0.279		
Abnormal LFTs at referral^d^	0.182	1.20 (0.455–3.162)	0.712		
ALP	0.006	1.006 (0.999–1.012)	0.099	1.004 (0.998–1.011)	0.190
ALT	0.002	1.002 (0.995–1.010)	0.574		
MCV^b^	0.005	1.005 (0.950–1.063)	0.856	0.971 (0.908–1.039)	0.399
Platelet count	0.000	1.000 (0.996–1.003)	0.786		
Bilirubin	0.025	1.026 (0.988–1.065)	0.187	0.999 (0.953–1.047)	0.966
AST^c^	0.003	1.003 (0.998–1.008)	0.215		
FIB4^c^	0.076	1.079 (0.996–1.169)	0.063		
AST/ALT ratio	0.733	2.081 (1.145–3.779)	0.016	1.984 (1.014–3.884)	0.046
APRI^c^	0.082	1.085 (0.997–1.182)	0.059		

BMI = Body Mass Index, U/w = units per week, CLD = Chronic Liver Disease, T2DM = Type 2 Diabetes Mellitus, ALT = Alanine aminotranferase, AST = Aspartate aminotransferase, MCV = Mean Corpuscular Volume, APRI = AST to platelet ratio index, ALP = Alkaline Phosphatase, OR = Odds Ratio

^a^Although *p* value for age was above 0.25 in univariable logistic regression, it was < 0.05 in correlation analysis with continuous ELF score, and is of clinical importance to investigate-so was included in this multivariable model

^b^Although *p* value for MCV was above 0.25 in univariable logistic regression, it was < 0.05 in correlation analysis with continuous ELF score, and so was included in this multivariable model

^c^Left out of multivariable analysis as would be affected by multi-collinearity with AST:ALT ratio, which was more highly significant in the univariate analysis

^d^Abnormal LFTs defined as raised transaminases or ALP + GGT. (Not including isolated hyperbilirubinaemia (Gilbert’s))

Out of the three indirect biomarkers of fibrosis investigated (FIB4, APRI and AST:ALT ratio), AST:ALT ratios trended towards being higher in the advanced fibrosis group (median 1.5, IQR 1.0–2.16), than in the group with lower ELF scores (median 1.26, IQR 0.8–1.6; *p* = 0.074). On univariate analysis the AST:ALT ratio did significantly predict advanced fibrosis based on ELF (OR 2.081 (95% CI 1.145–3.779), *p* = 0.016 (Table 2). On multivariable regression analysis, increasing AST:ALT ratio was the only variable significantly associated with ELF scores indicative of advanced fibrosis, when adjusted for age, alcohol intake, bilirubin, MCV, and ALP (OR 1.984, 95%CI (1.014–3.884), *p* = 0.046 (see Table 2).

### Factors associated with increasing ELF score

When literature derived risk factors for liver fibrosis were analysed against a continuous ELF score, there was no longer a significant association between the amount of alcohol consumption (U/w) and ELF score (*p* = 0.081) (Fig. 3a) and this was confirmed in multivariable regression analysis, both using continuous ELF (Table 3) and binary ELF scores </≥ 10.5 (Table 2). Alcohol intake was also analysed by grouping the units consumed per week into quartiles (0–79 U/w, 80–140 U/w, 141–280 U/w and 281 + U/w. There was no significant difference in ELF score between the quartiles either when ELF was analysed as a continuous score or using the 10.5 threshold. (See Additional file 1: Tables S1 and S2).

**Table 3:** Summary of multiple regression analysis of factors associated with continuous ELF score

Model	Unstandardized coefficients	Std. error	Standardized coefficients	Sig	95.0% Confidence interval for B	
	B		Beta		Lower bound	Upper bound
(Constant)	6.701	1.580		0.000	3.554	9.849
Age	0.024	0.009	0.278	0.013	0.005	0.042
Current alcohol intake (U/wk)	− 0.001	0.001	− 0.105	0.334	− 0.003	0.001
ALP	0.003	0.002	0.173	0.111	− 0.001	0.006
Bilirubin	0.018	0.012	0.170	0.146	− 0.006	0.042
MCV	0.014	0.016	0.094	0.374	− 0.018	0.046
AST:ALT ratio	0.115	0.170	0.077	0.502	− 0.224	0.454

(Dependent Variable: ELF score)

(ALP = Alkaline Phosphatase, MCV = Mean Corpuscular Volume, AST = Aspartate Aminotransferase, ALT = Alanine Aminotransferase)

ELF scores increased with increasing age (patients’ total age range 24–84) on univariate analysis (Fig. 3c), (*r* = 0.303, *p* = 0.002), and this was confirmed in multivariable analysis, when adjusted for alcohol intake, AST:ALT ratio, ALP, MCV and bilirubin (*p* = 0.013, 95% CI 0.005–0.042) (Table 3). For every 10-years increase in age, the ELF score increased by 0.24.

**Fig. 4:**
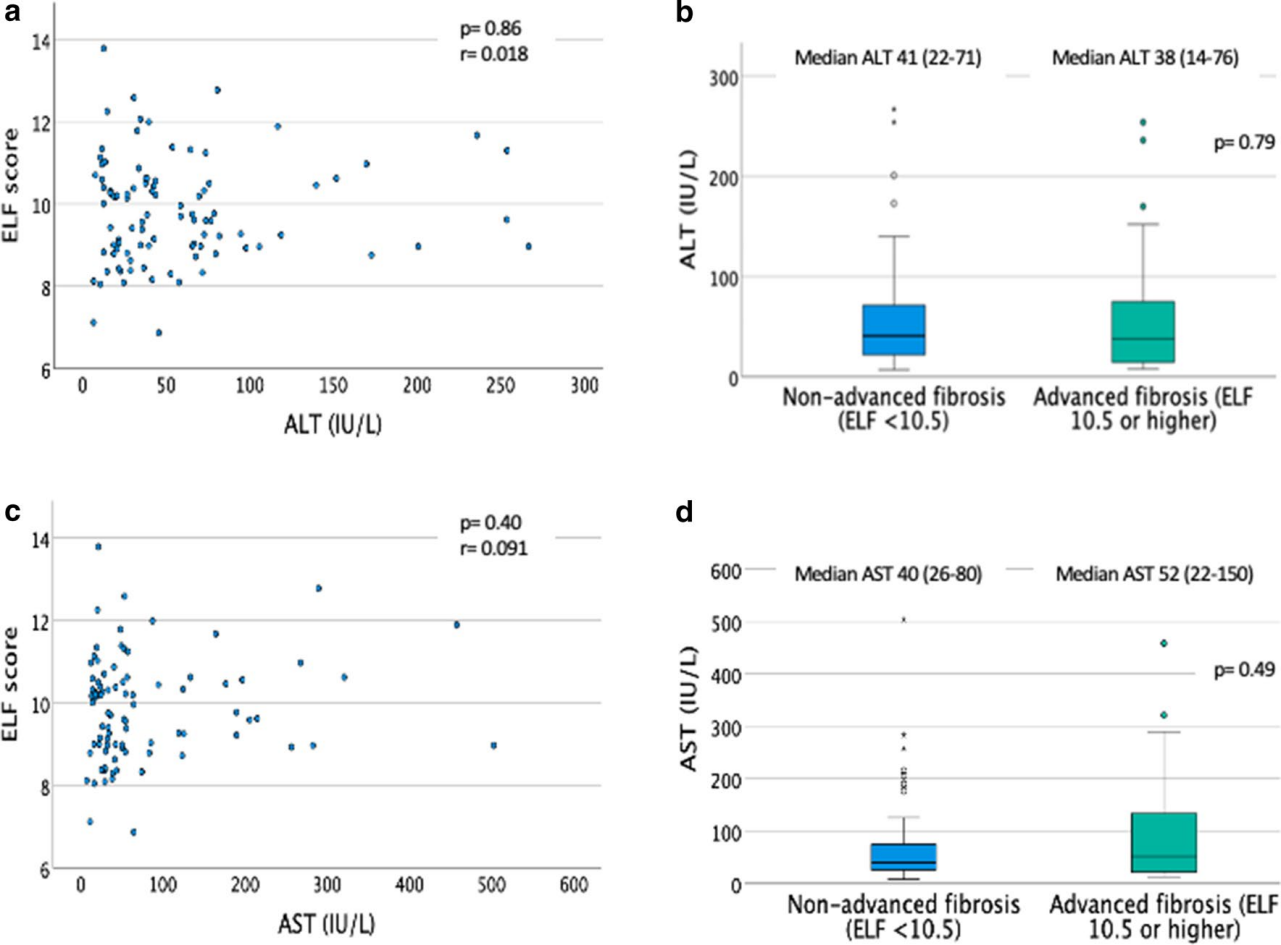
Influence of ALT and AST on binary and continuous ELF scores. **a** Scatter plot of ELF by ALT value (Spearman Rho correlation, with *p* value significance set at 0.05, r = correlation coefficient). **b** Boxplot of ALT by presence or absence of advanced fibrosis (ELF ≥ 10.5). Statistical test: Mann Whitney U, *p* value significance set at 0.05, ALT displayed with IQR (interquartile range). **c** Scatter plot of ELF by AST value (Spearman Rho correlation, with *p* value significance set at 0.05, r = correlation coefficient). **d** Boxplot of AST by presence or absence of advanced fibrosis (ELF ≥ 10.5). Statistical test: Mann Whitney U, *p* value significance set at 0.05, AST displayed with IQR (interquartile range)

ALT or AST were not associated with ELF score (either binary ELF of > or < 10.5, or continuous ELF (Fig. 4a–d and Table 2). Whilst AST:ALT ratio predicted advanced fibrosis when assessed using the 10.5 ELF threshold, a significant correlation was not seen between AST:ALT and continuous ELF score (r = 0.12, *p* = 0.27) (Additional file 1: Fig. S1a, b).

### FibroScan results

Of the 28 patients with ELF ≥ 10.5 who were offered a FibroScan appointment, only 18 attended, one of whom did not have a valid FibroScan reading, leaving 17 valid results (failure rate of 6%). The mean FibroScan value was 10.9 kPa (± 7.1 kpa), (total range of 4.2–25.3 kPa). Using a literature-derived threshold of 9.5 kPa for advanced fibrosis [15], 10/17 (58.8%) with ELF ≥ 10.5 had a FibroScan value < 9.5 kPa.

## Discussion

Over a quarter (28.3%) of patients with AUD presenting to hospital for a variety of reasons had an ELF score of ≥ 10.5, indicating the presence of advanced liver fibrosis. None of these patients had been assessed previously for liver fibrosis or referred to a liver specialist. All of them were at high risk of liver damage, with a current median alcohol consumption of 140 U/w, and history of excess alcohol consumption lasting more than 15 years, and yet none had been investigated for liver disease during their index presentation or at any time previously. Moreover, 76% of the cohort had presented to hospital on a median of four times per person over the preceding five years without a diagnosis of ArLD, indicating missed opportunities for detection and treatment of liver fibrosis in a high-risk population. Missed opportunities for recognising and assessing liver damage in primary care were not investigated in this study, but none of the patients in this study had been referred to hepatology services for assessment of liver disease prior to diagnosis in this study.

We found that LFTs were not a reliable predictor of advanced fibrosis, with 28% of patients who had ELF ≥ 10.5 having normal LFTs, in concordance with previous reports [7].

Whilst ELF scores were positively correlated with increasing age (*r* = 0.303, *p* = 0.002), there was no difference in the median ages of those with or without advanced fibrosis, as determined by ELF scores ≥ 10.5 or < 10.5 respectively. ELF score has been found to correlate with age in some [16, 17], but not all studies [18] and it is unclear how much of the reported correlation is due to the increased likelihood of advanced fibrosis being present in older patients [19]. McPherson et al. [20] studied the impact of age on the performance of a range of NIT (NFS, FIB4, AST:ALT ratio, but not ELF) in detecting advanced fibrosis (compared with biopsy) in patients with Non-Alcoholic-Fatty-Liver-Disease (NAFLD), and found that all the tests performed less well in people over the age of 65 (with an increase in false positive rates in this age group). They suggested the use of adjusted thresholds for diagnosing advanced fibrosis in this age range. Fagan et al. [16] found increased risk of false positives with ELF above the age of 45, concluding that caution needs to be taken in interpreting ELF scores in older age groups. Thiele et al. [14] also reported increased false positive ELF results in people over 60, and advised caution in interpreting ELF in the over 60 s. In contrast Parkes et al. [18] found no influence of age on ELF score in a cohort of patients with chronic hepatitis C (CHC) raising the possibility that age influences ELF score in NAFLD and ArLD but not in CHC.

The amount of alcohol consumed in U/w or duration of heavy drinking was not associated with ELF score in our cohort, and this was also the case in a large biopsy-controlled study of ELF in AUD by Thiele et al.[14] The same study also found that ALP was associated with increased ELF score, as observed in univariate analysis in this study, although not when adjusting for other factors in multivariable analysis.

Increasing AST:ALT ratio was the only other marker significantly associated with advanced fibrosis (ELF ≥ 10.5) in this study (OR 1.984, 95%CI 1.014–3.884, *p* = 0.046). Thiele et al. [14] found that AST:ALT had a Negative Predictive Value of 91% in a large biopsy-paired study and it may be that AST:ALT ratio could be used as a simple direct fibrosis test in addition to ELF in the assessment of advanced liver fibrosis in ArLD in a manner analogous to the combination of FIB4 and ELF in NAFLD [21] but this would require validation.

Whilst it has previously been reported that ELF scores may be influenced by inflammation [17, 22], we did not find any correlation between ELF score and ALT or AST, as markers of hepatic inflammation in this study, suggesting ELF was not influenced by inflammation in our cohort in keeping with findings of Thiele et al. study [14]. It must be noted, however, that patients with acute alcoholic hepatitis or acute liver injury from non-alcohol-related causes were excluded as ELF is not validated in these settings.

Limitations of this study include the lack of paired biopsies that would have provided a more robust reference standard assessment of liver fibrosis. However the use of non-invasive tests to assess liver fibrosis in in this study is representative of current clinical practice within the NHS and in many other countries, where patients presenting to hospital with AUD are not routinely biopsied, partly due to increasing recognition of the imperfections of biopsy as a test for liver fibrosis due to sampling error, inter and intra observer variability and the costs and hazards associated with biopsy [23, 24]. FibroScan was offered to all participants with ELF scores ≥ 10.5, but only 18/28 attended, of which valid readings were obtained for 17/18. Whilst FibroScan and ELF were discordant in 10/17 cases, FibroScan cannot be considered a robust reference standard measurement of fibrosis in ArLD, due to the impact of inflammation and alcohol on the accuracy of elastography. The small number of patients attending for FibroScan means that it is not possible to draw robust conclusions about the performance of FibroScan in this cohort. Furthermore, the poor attendance rate illustrates both the need to assess patients while they are inpatients, and the greater reliability of using a blood test to assess fibrosis that can be incorporated in routine investigations.

In common with routine practice, we relied on patients’ self-reported alcohol intake extracted from clinical records, an approach that is likely to be inaccurate. Unfortunately, it is not local routine practice to obtain AUDIT scores but these would provide additional valuable information about drinking behaviour. Fibrosis was assessed using a single ELF test at the start of the patients’ hospital admission. Although liver stiffness as measured by FibroScan reduces significantly on withdrawal of alcohol [25–27], a study of ten patients found that there was no significant difference in the ELF scores recorded from intoxicated patients when re-tested two weeks after alcohol withdrawal [28] but the impact of drinking on ELF score needs further investigation.

Overall, this study has highlighted the missed opportunities for detecting liver fibrosis in at-risk patients in a hospital setting. Alcohol use disorder must be viewed as a multimorbid condition with psycho-social morbidity and the potential to damage every organ in the body. However, alcohol related liver disease accounts for much of the mortality and costs of drinking and accurate and relatively inexpensive blood tests are now available that permit detection of liver damage in all those at risk. It could be argued that there is no longer any excuse to miss the diagnosis of liver fibrosis in patients presenting to hospital with AUD. Whilst people with AUD encompass some of the more socially disadvantaged members of society that may find engaging with routine health services difficult, it is imperative that all opportunities to detect liver fibrosis should be taken especially on those occasions when they present to hospital with complications of AUD or other conditions.

BSG guidance now recommends non-invasive fibrosis testing for people with high-risk alcohol intake (> 35 U/w in women, > 50 U/w in men) [7] with either FibroScan or ELF. This study emphasises the importance of implementing this guidance and incorporating it into hospital guidelines in emergency departments and in alcohol care teams [29] to improve the detection of advanced fibrosis in people with AUD.

## Supplementary Information

**Additional file 1. Table S1**: Multiple logistic regression analysis to investigate for effect of alcohol unit quartiles on presence or absence of advanced fibrosis (as per ELF ≥ 10.5). **Table S2**: Multiple linear regression analysis to investigate for effect of alcohol unit quartiles on continuous ELF score. **Fig. S1**: Influence of AST:ALT ratio on binary and continuous ELF scores. **a** Scatter plot of ELF by AST:ALT ratio (Spearman Rho correlation, with *p* value significance set at 0.05, r = correlation coefficient). **b** Boxplot of AST:ALT ratio by presence or absence of advanced fibrosis (ELF ≥ 10.5). Statistical test: Mann Whitney U, *p* value significance set at 0.05, AST:ALT ratio displayed with IQR (interquartile range).

## Data Availability

On publication of this article, the dataset will be made available from the corresponding author on reasonable request.

## Abbreviations

ArLD: Alcohol related liver disease
CLD: Chronic liver disease
AUD: Alcohol use disorder
NIT: Non-invasive test
ELF: Enhanced liver fibrosis
ALT: Alanine aminotransferase
AST: Aspartate aminotransferase
ALP: Alkaline phosphatase
FIB4: Fibrosis 4 score
APRI: AST to platelet ratio index
BMI: Body mass index
T2DM: Type II diabetes mellitus
OR: Odds ratio
CI: Confidence interval
U/w: Units per week
BSG: British Society of Gastroenterology
