# Supplementary material for: Uncovering unsuspected advanced liver fibrosis in patients referred to alcohol nurse specialists using the ELF test
Source: BMC Gastroenterol. 2021 Mar 31;21:143. doi: 10.1186/s12876-021-01728-2 (PMC8011169; doi:10.1186/s12876-021-01728-2)

**Supplementary Materials for Uncovering unsuspected advanced liver fibrosis in patients referred to alcohol nurse specialists using the ELF test**

Freya Rhodes^1*^, Sara Cococcia^1,2*^, Jasmina Panovska-Griffiths^3,4^, Sudeep Tanwar^1,5^, Rachel H Westbrook^1^, Alison Rodger^4^, William M. Rosenberg^1^

^1^Institute for Liver and Digestive Health, UCL Division of Medicine, Royal Free Campus, London, UK

^2^First Department of Internal Medicine, San Matteo Hospital Foundation, University of Pavia, Pavia, Italy

^3^Department of Applied Health Research, University College London, UK

^4^Institute for Global Health, University College London, UK

^5^ Barts Health NHS Trust, London, United Kingdom

***Joint 1^st^ authors**

**CORRESPONDENCE:**

Professor William M Rosenberg: [w.rosenberg@ucl.ac.uk](mailto:w.rosenberg@ucl.ac.uk)

Address: Institute for Liver and Digestive Health, Division of Medicine, University College London, Royal Free Campus, Rowland Hill Street, Hampstead, London NW3 2PF, United Kingdom.

ORCID ID: 0000-0002-2732-2304

**Keywords**: (Liver cirrhosis), (liver diseases, alcoholic), (non-invasive test), (Alcohol Use Disorder), (Enhanced Liver Fibrosis test).

**SUPPLEMENTARY TABLES**

**Supplementary Table 1:**

|  |  |  |  |  |  |  |  |  |
| --- | --- | --- | --- | --- | --- | --- | --- | --- |
| **Quartiles of alcohol units/week** | **B** | **S.E.** | **Wald** | ***df*** | **P value** | **OR** | **95% C.I. for OR** | |
|  |  |  |  |  |  |  | **Lower** | **Upper** |
| 80-140 U/Wk (n=27) | -.963 | .606 | 2.525 | 1 | .112 | .382 | .116 | 1.252 |
| 141-280 U/Wk (n=35) | -1.129 | .580 | 3.793 | 1 | .051 | .323 | .104 | 1.007 |
| 281-840 U/Wk (n=10) | -2.110 | 1.134 | 3.464 | 1 | .063 | .121 | .013 | 1.118 |
| Constant (0-79 U/Wk) (n=23) | -.087 | .417 | .043 | 1 | .835 | .917 |  |  |

**Supplementary Table 2:**

| **Model** | **Unstandardized Coefficients** |  | **Standardized Coefficients** | **Sig.** | **95.0% Confidence Interval for B** | |
| --- | --- | --- | --- | --- | --- | --- |
|  | **B** | **Std. Error** | **Beta** |  | **Lower Bound** | **Upper Bound** |
| (Constant) | 9.213 | .700 |  | .000 | 7.823 | 10.604 |
| 0-79 U/Wk (n=23) | 1.270 | .743 | .435 | .091 | -.205 | 2.745 |
| 80-140 U/Wk (n=27) | .416 | .738 | .148 | .575 | -1.050 | 1.881 |
| 141-280 U/Wk (n=35) | .501 | .730 | .191 | .494 | -.948 | 1.950 |
| 281-840 U/Wk (n=10) | .007 | .799 | .002 | .993 | -1.579 | 1.592 |
| Dependent Variable: ELF score | | | | | | |

**Supplementary Fig. 1 A, B:**


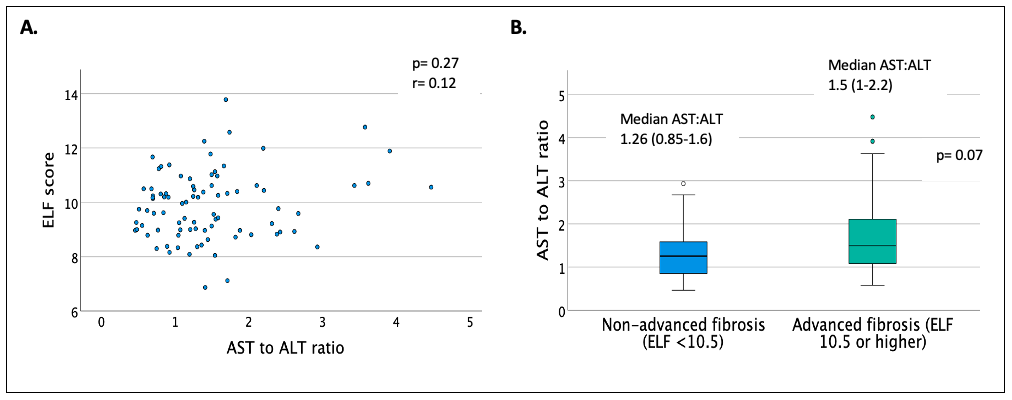

Supplement: Supplementary file 1 — Additional file 1. Table S1: Multiple logistic regression analysis to investigate for effect of alcohol unit quartiles on presence or absence of advanced fibrosis (as per ELF ≥ 10.5). Table S2: Multiple linear regression analysis to investigate for effect of alcohol unit quartiles on continuous ELF score. Fig. S1: Influence of AST:ALT ratio on binary and continuous ELF scores. a Scatter plot of ELF by AST:ALT ratio (Spearman Rho correlation, with p value significance set at 0.05, r = correlation coefficient). b Boxplot of AST:ALT ratio by presence or absence of advanced fibrosis (ELF ≥ 10.5). Statistical test: Mann Whitney U, p value significance set at 0.05, AST:ALT ratio displayed with IQR (interquartile range). [file 12876_2021_1728_MOESM1_ESM.docx]
